# Bone Marrow Mesenchymal Stromal Cells Stimulate Skeletal Myoblast Proliferation through the Paracrine Release of VEGF

**DOI:** 10.1371/journal.pone.0037512

**Published:** 2012-07-16

**Authors:** Chiara Sassoli, Alessandro Pini, Flaminia Chellini, Benedetta Mazzanti, Silvia Nistri, Daniele Nosi, Riccardo Saccardi, Franco Quercioli, Sandra Zecchi-Orlandini, Lucia Formigli

**Affiliations:** 1 Department of Human Anatomy, Histology and Forensic Medicine, University of Florence, Florence, Italy; 2 Department of Hematology, Cord Blood Bank, Careggi Hospital, University of Florence, Florence, Italy; 3 National Institute of Optics (INO), National Research Council (CNR), Sesto Fiorentino, Florence, Italy; University of Medicine and Dentistry of New Jersey, United States of America

## Abstract

Mesenchymal stromal cells (MSCs) are the leading cell candidates in the field of regenerative medicine. These cells have also been successfully used to improve skeletal muscle repair/regeneration; however, the mechanisms responsible for their beneficial effects remain to be clarified. On this basis, in the present study, we evaluated in a co-culture system, the ability of bone-marrow MSCs to influence C2C12 myoblast behavior and analyzed the cross-talk between the two cell types at the cellular and molecular level. We found that myoblast proliferation was greatly enhanced in the co-culture as judged by time lapse videomicroscopy, cyclin A expression and EdU incorporation. Moreover, myoblasts immunomagnetically separated from MSCs after co-culture expressed higher mRNA and protein levels of Notch-1, a key determinant of myoblast activation and proliferation, as compared with the single culture. Notch-1 intracellular domain and nuclear localization of Hes-1, a Notch-1 target gene, were also increased in the co-culture. Interestingly, the myoblastic response was mainly dependent on the paracrine release of vascular endothelial growth factor (VEGF) by MSCs. Indeed, the addition of MSC-derived conditioned medium (CM) to C2C12 cells yielded similar results as those observed in the co-culture and increased the phosphorylation and expression levels of VEGFR. The treatment with the selective pharmacological VEGFR inhibitor, KRN633, resulted in a marked attenuation of the receptor activation and concomitantly inhibited the effects of MSC-CM on C2C12 cell growth and Notch-1 signaling. In conclusion, this study provides novel evidence for a role of MSCs in stimulating myoblast cell proliferation and suggests that the functional interaction between the two cell types may be exploited for the development of new and more efficient cell-based skeletal muscle repair strategies.

## Introduction

Skeletal muscle has a robust capacity of repair/regeneration in response to injury or disease, relying in large part upon the presence of a population of skeletal myoblast precursors, the satellite cells, whose activation, re-entry the cell cycle and differentiation require signals emanated by damaged fibers and infiltrating inflammatory cells [1], [2]. However, these cells are relatively scarce within the skeletal muscle tissue, comprising about 1% to 5% of the total muscle nuclei, and are not able to be recruited in large number at the site of tissue damage. Therefore, during disease or other adverse conditions, the injured muscle is replaced by a fibrotic scar which typically accompanies the muscle decline and compromises its function. Because of their features, satellite cells represent the obvious cellular candidate to target in muscle regenerative medicine. There are, in fact, several studies in the literature focusing on the identification of factors improving the growth and regenerative potential of these cells in their microenvironment [3] and there is a number of examples of satellite cell transplantation for skeletal muscle regeneration [4]–[6]. However, the full potential of satellite-cell therapy is affected by several limitations, including the high heterogeneity of this cell population [7], the loss of their myogenic potential upon *in vitro* expansion [6] and the predetermination dependent from the source of muscle fibers [8], [9]. This has shifted the attention to other cell sources of non-myogenic origin as additional candidates for skeletal muscle repair/regeneration. In this field, transplantation of mesenchymal stromal cells (MSCs) in animal models of myopathies, including the ischemic, atrophic and dystrophic muscle, has been shown to remarkably improve the functional recover of the injured tissue [10]–[12]. MSCs are a rare population of cells that can be isolated from the bone marrow, adipose tissue and many other regions of the body, rapidly expanded *ex vivo* and utilized for experimental and clinical studies. They have the potential to acquire lineage of any-mesenchymal-derived tissue *in vitro.* Despite their plasticity, the participation of MSCs to new skeletal muscle fiber formation is controversial [10], [13], [14]; emerging evidence from a variety of injured adult tissues indicates that their therapeutic effects occur without evidence of long-term tissue engraftment [15]–[18]. Indeed, the functional improvements in injured tissue seem to be primarily due to the secretion by the transplanted MSCs of cytokines and growth factors with multiple effects on the injured tissue, including modulation of inflammation and immune reaction, positive remodeling, angiogenesis and protection from apoptosis [19]–[21]. We have recently reported in a co-culture system that MSCs support proliferation of neonatal cardiomyocyte precursors through combined paracrine/juxtacrine mechanisms [22], suggesting the potential ability of these cells to determine the fate of local stem cells and augment the endogenous repair of the damaged tissues.

In the present study, we further explored this issue by investigating whether MSCs influenced the *in vitro* behavior of skeletal myoblasts. Using a co-culture system of bone-marrow MSCs and C2C12 cells, we provided novel experimental evidence that MSCs could interact with the skeletal myoblasts by stimulating their proliferation potential and underscored the molecular and cellular mechanisms involved.

## Materials and Methods

### Ethics Statement

All animal manipulations were carried out according to the European Community guidelines for animal care (DL 116/92, application of the European Communities Council Directive of 24 November 1986; 86/609/EEC) and approved by the Committee for Animal Care and Experimental Use of the University of Florence. The ethical policy of the University of Florence conforms to the Guide for the care and use of laboratory animals of the U.S. National Institutes of Health (NIH Publication No. 85–23, revised 1996; University of Florence assurance No. A5278-01). The protocols were communicated to local authorities and to Italian Ministry of the Health; according to the Italian law (Art.7/D.lgs 116/92) such procedure doesn’t require Ministry authorization. The animals were housed with free access to food and water and maintained on a 12 h light/dark cycle at 22C room temperature (RT). All efforts were made to minimize the animal suffering and the number of animals sacrified. Animals were killed by decapitation.

### Cell Culture

*Murine C2C12 skeletal myoblasts* obtained from American Type Culture Collection (ATCC, Manassas, VA, USA), were grown in Dulbecco’s modified Eagle’s medium (DMEM) supplemented with 10% fetal bovine serum (FBS), 1% penicillin/streptomycin (Sigma, Milan, Italy) at 37°C in a humidified atmosphere of 5% CO_2_ till reaching 80% confluence and then they were shifted in differentiation medium (myoblast DM), containing 2% horse serum (HS, Sigma) for 12, 24, 48, 72 h and 5 days.

*Mouse bone marrow mesenchymal stromal cells* (MSCs) were isolated from femura and tibiae of male C2F1 mice, following the Dobson procedure [23], expanded *in vitro* and characterized as reported previously [22]. These cells were cultured in myoblast DM for 24 h and, in same experiments, the culture medium (MSC- derived conditioned medium, MSC-CM) was harvested and used for culturing C2C12 myoblasts for 24 h to assess MSC paracrine effects.

*Transgenic bone marrow green fluorescent protein (GFP)-labeled MSCs* were isolated from male GFP transgenic Lewis rats (RRRC, Missouri, USA), expanded and characterized as described previously [24]. GFP-labeled MSCs were analyzed for green fluorescence intensity at different passages in culture as well as for the expression of particular cell surface molecules using flow cytometry procedures: CD45-CyChromeTM, CD11b-FITC (in order to quantify hemopoietic-monocytic contamination), CD90-PE, CD73-PE, CD44-PE (BD Pharmingen, San Diego, CA, USA).

#### Co-cultures of C2C12 myoblasts and MSCs

C2C12 cells were co-cultured with mouse MSCs or GFP-labeled MSCs at a 2∶1 ratio for different times, from 6 h up to 5 days in myoblast DM. Mouse MSCs in the co-cultures were identified by labeling with the fluorescent VybrantTM Dil Cell-Labeling solutions (Molecular Probes, Eugene, OR, USA) according to the manufacturer’s instructions.

### Cell Treatments

C2C12 cells were treated with an ATP competitive inhibitor of VEGFR tyrosine kinase activity, KRN633 (IC50 = 170 nM, Santa Cruz Biotechnology, Santa Cruz, CA, USA) for 24 h, or with a specific γ-secretase inhibitor, DAPT for 24 h to inhibit Notch-1 activation (5 µM, stock 5 mM in dymethil sulfoxide, DMSO, 0.1%, Sigma), or with soluble Vascular Endothelial Growth Factor, VEGF (2 ng/ml and 20 ng/ml, Sigma) for 24 h, 48 h and 5 days.

### Time-lapse Videomicroscopy

The dynamic behavior of the single cultures and the co-cultures were analyzed for 72 h by time-lapse videomicroscopy (1 frame/min, exposure time 0.5 s) using an inverted phase-contrast and fluorescence microscope (Nikon, Tokyo, Japan) equipped with a 10X objective and a cooled video camera equipped with a motorized filter wheel and its dedicated digital recording software (Chroma CX3, DTA, Cascina, Italy). Quantitative analysis of the cell growth was performed by direct cell counting and expressed as percentage increase.

### EdU (5-ethynyl-2′-deoxyuridine) Incorporation Assay

Cell proliferation was evaluated by the EdU proliferation assay using the fluorescent Click-iT® EdU Cell Proliferation Assay (Invitrogen Life Technologies, Grand Island, NY, USA) according to manufacturer’s instructions. This assay is based on the incorporation of the pyrimidine analogue EdU in place of thymidine into newly synthesized DNA of replicating cells. Briefly, cells grown on glass coverslips were incubated in the presence of the provided solution of 10 µM EdU for 24 h. After that, the cells were washed with PBS, fixed with 0.5% buffered paraformaldehyde (PFA) for 10 min at RT, permeabilized with cold acetone for 3 min and then incubated with the Alexa Fluor 488 EdU detection solution for 30 min at RT, protected from light. After washing, the coverslips containing the labeled cells were mounted with an antifade mounting medium (Biomeda Gel mount, Electron Microscopy Sciences, Foster City, CA) and observed under a confocal Leica TCS SP5 microscope (Leica Microsystems, Mannheim, Germany). The number of the cells with EdU positive nuclei was evaluated in 10 random 140.000 µm^2^ square microscopic fields (63X ocular) in each cell preparation and expressed as percentage of the total cell number.

### Morphological Analyses

#### Confocal immunofluorescence

The cells, grown on glass coverslips were fixed with 0.5% buffered PFA for 10 min at RT. After 3 min permeabilization with cold acetone, the fixed cells were blocked with 0.5% Bovine Serum Albumine (BSA) and 3% glycerol in PBS for 20 min and incubated overnight at 4°C with the following antibodies: rabbit polyclonal anti-cyclin A (1∶100; Santa Cruz Biotechnology, Santa Cruz, CA, USA), mouse monoclonal anti-myogenin (1∶50; Sigma), rabbit monoclonal anti-Notch-1 (1∶200; Abcam, Cambridge, UK), rabbit polyclonal anti-Hes1 (1∶300; Millipore, Milan, Italy) and goat polyclonal anti-VEGFR2 (Flk-1, 1∶50; Abcam). The immunoreactions were revealed by incubation with specific anti-mouse, anti-rabbit or anti-goat Alexa Fluor 488 or 568-conjugated IgG (1∶200; Molecular Probes) for 1 h at RT. Negative controls were carried out by replacing the primary antibodies with non immune serum; cross-reactivity of the secondary antibodies was tested in control experiments in which primary antibodies were omitted. After washing, the coverslips containing the immunolabeled cells were mounted with an antifade mounting medium (Biomeda Gel mount) and observed under a confocal Leica TCS SP5 microscope (Leica Microsystems) equipped with a HeNe/Ar laser source for fluorescence measurements and with differential interference contrast (DIC) optics. Observations were performed using a Leica Plan Apo 63X/1.43NA oil immersion objective. Series of optical sections (1024×1024 pixels each; pixel size 204.3 nm), were taken through the depth of the cells at intervals of 0.4 µm. Images were then projected onto a single ‘extended focus’ image. When needed, a single optical fluorescent section and DIC images were merged to view the precise distribution of the immunostaining. Quantification of cyclin A, myogenin and Notch-1 expression was performed on digitized images by counting the number of positive cells over the total cell number. Densitometric analysis of the intensity of Hes-1 fluorescent signal was performed on digitized images using ImageJ software (http://rsbweb.nih.gov/ij) in 20 regions of interest (ROI) of 100 µm^2^ for each confocal stack (at least 10).

#### Phase contrast microscopy

Myotube formation was assayed in 10 random 1.200.000 µm^2^ optical square fields (20X) under an inverted phase contrast microscopy (Nikon Diaphot 300) in each cell preparation.

### Flow Cytometry

Flow cytometry assay was used to distinguish the two cell populations on the basis of their cell surface antigen expression. To this aim, MSCs and C2C12 cells recovered from flask by trypsin- Ethylenediaminetetraacetic acid (EDTA) treatment, were re-suspended in flow cytometry buffer consisting of CellWASH (0.1% sodium azide in PBS; Becton Dickinson, San Jose, CA, USA) with 2% FBS and incubated with FITC-or PE- conjugated monoclonal antibodies (BD Pharmingen, San Diego, CA, USA) against myoblastic (CD34) and mesenchymal (CD73) cell marker [22], [25]; 7-aminoactinomycin AAD (7-AAD, BD Pharmingen) was added in order to exclude dead cells from the analysis. Flow cytometric acquisition was performed by collecting 10^4^ events on a FACScalibur (Becton Dickinson) instrument and data were analyzed on DOT-PLOT bi-parametric diagrams using CELL QUESTPRO software (Becton Dickinson, San Jose, CA, USA) on Macintosh PC.

### Immunomagnetic Cell Separation

C2C12 myoblasts and MSCs were separated after 24 h co-culture using MACS micro beads technology (Miltenyi Biotec, Bologna, Italy). In particular, the co-cultured cells were recovered by trypsin-EDTA treatment resuspended in Buffer containing 0.5% BSA and 2 mM EDTA and incubated with CD73 or CD34 PE-conjugated antibody (BD Pharmingen) following manufacturer’s instructions. Cells were then incubated with anti-PE MicroBeads and separated on MS MACS column following manufacturer’s instructions (Miltenyi Biotec). The positive and negative cell fractions were then analyzed by flow cytometry and processed for Western Blotting and RT-PCR analysis. C2C12 cells in single culture were subjected to the same treatments of those in co-culture and used as control.

### Total RNA Extraction and Reverse Transcription (RT)-PCR

Expression levels of mRNA of Notch-1, Jagged-1 and Hes-1 were assayed by RT-PCR. Total RNA was isolated by extraction with TRIzol Reagent (Invitrogen), according to the manufacturer’s instructions. One µg of total RNA were reverse transcribed and amplified with SuperScript One-Step RT-PCR System (Invitrogen). The following mouse gene-specific primers were used: Notch-1 (NM_008714.3), forward 5′-GTC CCA CCC ATG ACC ACT AC-3′ and reverse 5′-CCT GAA GCA CTG GAA AGG AC-3′; Jagged-1 (NM_013822.4), forward 5′-CAG GAC ACA CAA CTC GGA AG-3′ and reverse 5′-CCA GCC AAC CAC AGA AAC TAC-3′; Hes-1 (NM_ 008235), forward 5′-AAT TTG CCT TTC TCA TCC CCA-3′ and reverse 5′- CAG TCA CTT AAT ACA GCT CTC-3′ β-actin (NM_007393.3), forward 5′-ACT GGG ACG ACA TGG AGA AG-3′ and reverse 5′- ACC AGA GGC ATA CAG GGA CA-3′. β-actin mRNA was used as internal standard. Blank controls, consisting in no template (water), were performed in each run. PCR products were separated by electrophoresis on a 2% agarose gel and the ethidium bromide-stained bands were quantified by densitometric analysis by using the Scion Image Beta 4.0.2 image analysis program (Scion Corp., Frederick, MD, USA). β-actin normalization was performed for each result.

### Western Blotting

Cells were resuspended in appropriate volume of cold lysis buffer: 10 mmol/L Tris/HCl, pH 7.4, 10 mmol/L NaCl, 1.5 mmol/L MgCl_2_, 1% Triton X-100, 2 mmol/L Na_2_EDTA, added with 10× Sigmafast Protease Inhibitor cocktail tablets (Sigma). Upon centrifugation at 13.000 g for 15 min at 4°C, the supernatants were collected and the total protein content was measured spectrophotometrically using micro-BCA™ Protein Assay Kit (Pierce, IL, USA). Forty µg of total proteins were electrophoresed by SDS-PAGE (200V, 1 h) using a denaturating 7.6% polyacrylamide gel and blotted onto nitrocellulose membranes (Amersham, Cologno Monzese, Italy; 150V, 1 h). The membranes were blocked with PBS containing 0.1% Tween (Sigma) and 5% bovine serum albumin (AT-PBS) (Sigma) for 1 h at RT and incubated overnight at 4°C with rabbit monoclonal anti-Notch-1 antibody (1∶2000 in AT-PBS; Abcam) goat polyclonal anti-Jagged-1 antibody (1∶1000 in AT-PBS; Santa Cruz Biotechnology), rabbit polyclonal anti-Hes-1 (1∶20.000 in AT-PBS Millipore) and goat polyclonal anti-VEGFR2 (Flk-1) (1∶1000 in AT-TBS; Abcam). Membranes were also immunostained with rabbit polyclonal anti-β-actin antibody (1∶20.000 in AT-PBS; Sigma), assuming β -actin as control invariant protein. After washing, the membranes were incubated with peroxidase-labeled anti-rabbit or anti-goat antibodies (1∶15.000 in AT-PBS; Vector, Burlingame, CA, USA) for 1 h at RT and the immunoreactivity was detected by the ECL chemiluminescent substrate (Amersham). Densitometric analysis of the bands was performed using Scion Image Beta 4.0.2 image analysis software (Scion Corp.) and the values normalized to β-actin.

### Immunoprecipitation

The cells were lysed as reported previously [26]. One mg of whole cell extract was pre-cleared by Protein A/G Plus-Agarose (Santa Cruz Biotechnology) for 1 h at 4°C. After centrifugation, the supernatants were collected and incubated overnight at 4°C with gentle inverting, with 4 µg of goat polyclonal anti-VEGFR2(Flk-1) antibody (Abcam). Then the samples were re-incubated with Protein A/G Plus-Agarose for 2 h at 4°C and precipitated by centrifugation. Complexes were subjected to electrophoresis and blotted with mouse monoclonal anti-phospho-tyrosine antibody (1∶1000 in AT-TBS; Sigma) and then re-probed with anti- VEGFR2(Flk-1) antibody.

### Cytokine/growth Factor Assay

Interleukin (IL)-15, IL-18, basic fibroblast growth factor (bFGF), leukemia inhibiting factor (LIF), macrophage colony stimulating factor (M-CFS), interferon-γ-induced monokine (MIG), macrophage inflammation protein (MIP)-2 and VEGF-1 levels were measured in MSC-CM by using Bio-Plex Pro Mouse Cytokine assay (Bio Rad Laboratories Inc., Hercules, CA, USA) following the manufacturer’s instructions essentially as described previously [22].

### Statistical Analysis

Data were reported as mean ± SEM and the statistical significance was determined by one-way ANOVA and Newman-Keuls multiple comparison test or Student’s *t* test. A *p* value ≤0.05 was considered significant. Calculations were performed usingGraphPad Prism software (GraphPad, San Diego, CA, USA).

## Results

### MSCs Stimulate C2C12 Cell Proliferation

**Figure 1:**
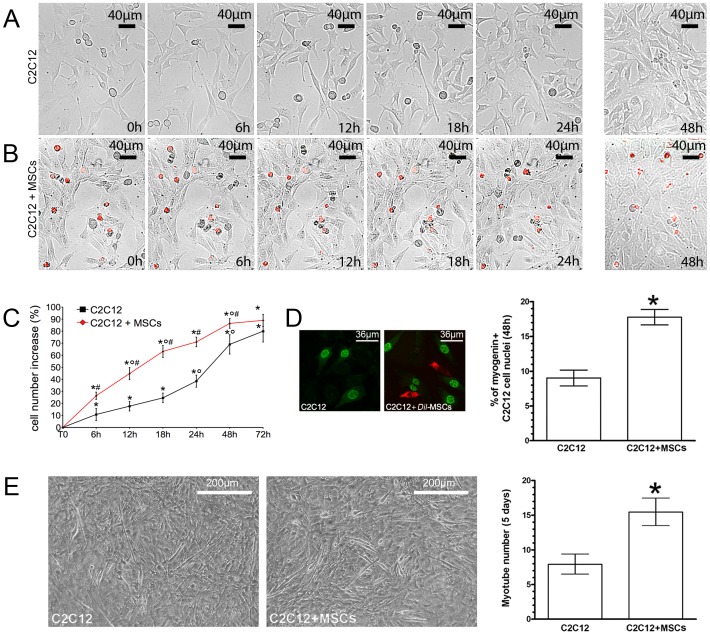
Effects of MSCs on myoblast cell growth and differentiation. Time lapse videoimaging of C2C12 cells in single (A) and co-culture with *Dil* (red)-labeled MSCs (B). Quantitative analyses of C2C12 cell proliferation in the different experimental conditions (C). Note that the presence of MSCs greatly enhances myoblast proliferation. D) Confocal immunofluorescence to detect myogenin (green) expression in C2C12 cells in single and co-culture with *Dil*-labeled MSCs; the quantitative analysis is reported in the corresponding histogram. E) Phase contrast microscopy showing myotube formation in single and co-cultured C2C12 cells; the quantitative analysis is reported in the corresponding histogram. The results of these experiments clearly show that myogenin is up-regulated in the co-cultured C2C12 cells and that myoblasts are the only cell type to contain myogenin^+^ nuclei. Also their tendency to fuse into multinucleated myotube is greater in the presence of MSCs. Data represent the results of at least three independent experiments with similar results. C: *p<0.05 versus T0; °*p<0.05* versus the earlier time points; #*p<0.05* versus single culture. D, E: *p<0.05.

**Figure 2:**
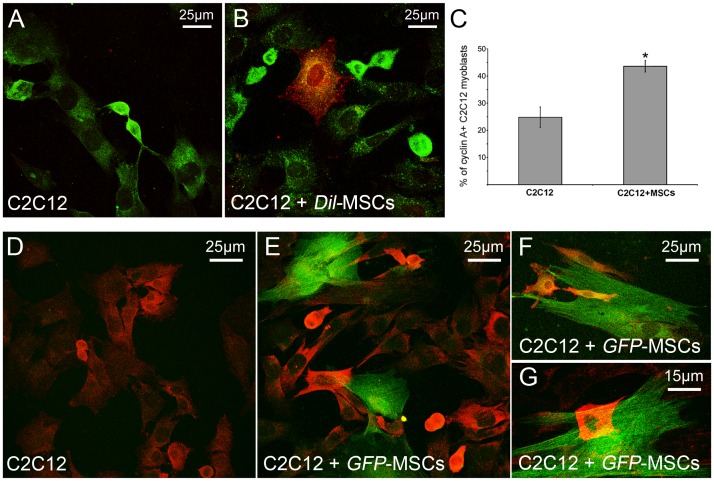
Assessment of myoblast cell proliferation by cyclin A expression. C2C12 cells in single and co-culture with *Dil*(red)- or *GFP*(green)-labeled MSCs for 24 h were incubated with specific antibodies against cyclin A (green, A,B; red, D-G) and observed by confocal microscopy. Notably, the cells with the higher immunofluorescence intensity are those located in close contact with MSCs. C) Quantitative analysis of the number of cells positive for cyclin A. Data represent the results of at least three independent experiments with similar results. *p<0.05.

To examine whether myoblast proliferation was modulated by interactions with MSCs, we used a model of direct co-culture of native C2C12 cells and MSCs marked with *Dil* (*Dil*-MSCs), in order to distinguish the behavior of the two cell types. Time-lapse videoimaging and cell counting showed that the co-presence of MSCs significantly enhanced C2C12 cell proliferation, since the early times of co-cultures (from 6 h up to 48 h) (Fig. 1A–C). MSCs also proliferated, although at a markedly lower rate than the adjacent C2C12 cells (1% increase after 24 h), and established transient cell-cell-contacts with C2C12 cells (Fig. 1B). In the co-culture, myoblast proliferation gradually decreased after 24 h and became negligible after 72 h; whereas in the single culture, the cells continued to actively proliferate up to 48 h (Fig. 1C). Increased myoblast proliferation in the co-culture with MSCs was associated with increased myoblast differentiation, as judged by the analysis of the expression of myogenin, a key myogenic differentiation factor, (Fig. 1D), and by the tendency of the cells to form multinucleated myotubes at longer time points (5 days) (Fig. 1E). MSCs in the co-culture failed to express myogenic markers, such as myogenin, or fuse with differentiating myoblasts (data not shown), indicating that these cells were unable to trans-differentiate along the myoblastic lineage in our experimental conditions. The ability of MSCs to potentiate C2C12 myoblast proliferation was further confirmed by the findings of increased EdU incorporation (83.3±8% and 63.3±5% of the myoblasts resulted EdU positive in the co-cultures and single culture, respectively, *p*<0.05 by Student’s Test) and cyclin A nuclear and cytoplasmic expression in the myoblasts co-cultured for 24 h with either *Dil*- (Fig. 2A–C) or *GFP*-MSCs (Fig. 2 D–G) as compared to those in the single culture.

**Figure 3:**
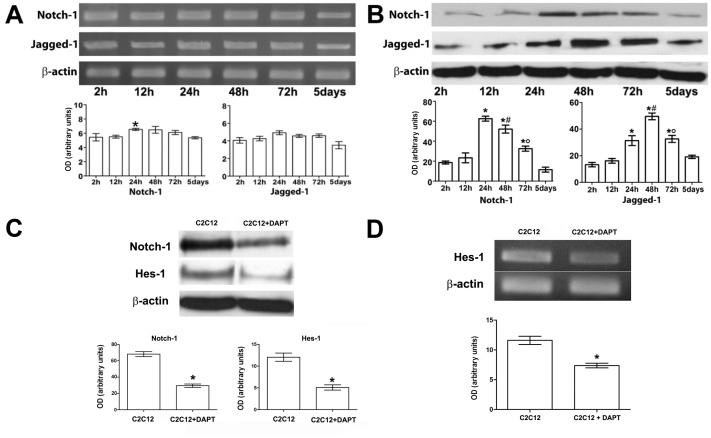
Notch-1 patway in differentiating C2C12 myoblasts. A,B) mRNA and protein expression of Notch-1 receptor and its ligand, Jagged 1, evaluated by RT-PCR (A) and Western Blotting (B) in C2C12 cells in single culture; densitometric analysis of the bands normalized to β-actin is reported in the histograms. C,D) Hes-1 expression in cells treated with 5 µM DAPT, a pharmacological inhibitor of Notch-1 activation. Note that the inhibition of Notch-1 expression (C) is capable of reducing the expression of Hes-1 at the mRNA (D) and protein levels (C). Data represent the results of at least three independent experiments with similar results. A, B, *p<0.05 versus 2 h; #*p<0.05* versus 24 h; °p<0.05 versus 48 h.C,D, *p<0.05.

To unveil the molecular mechanisms underlying the stimulatory effects of MSCs on C2C12 cell proliferation, we investigated the involvement of Notch-1, a key determinant of myoblast activation and skeletal muscle regeneration [27]. We first showed that Notch-1 and its ligand Jagged-1 were expressed at high levels in differentiating C2C12 cells as judged by RT-PCR and Western blotting; in particular, while the levels of Notch-1 and Jagged-1 mRNA remained practically costant during the whole period of differentiation, the expression of the corresponding proteins reached the maximal values in the early stages (24 h for Notch-1 and 48 for Jagged-1), in coincidence with the higher proliferative activity (Fig. 3A,B). Although the reasons for this discrepancy is not known, it is possible that the regulation of Notch-1 pathway may follow distint pathway at the mRNA and protein levels in our cell system. We also showed that the treatment of C2C12 cells with a specific pharmacological inhibitor of Notch-1 activation, DAPT, was able to affect the expression of Hes-1 at the mRNA and protein levels (Fig. 3C,D; Fig. 5E,H), thus confirming its role as a target gene of Notch-1 action.

**Figure 4:**
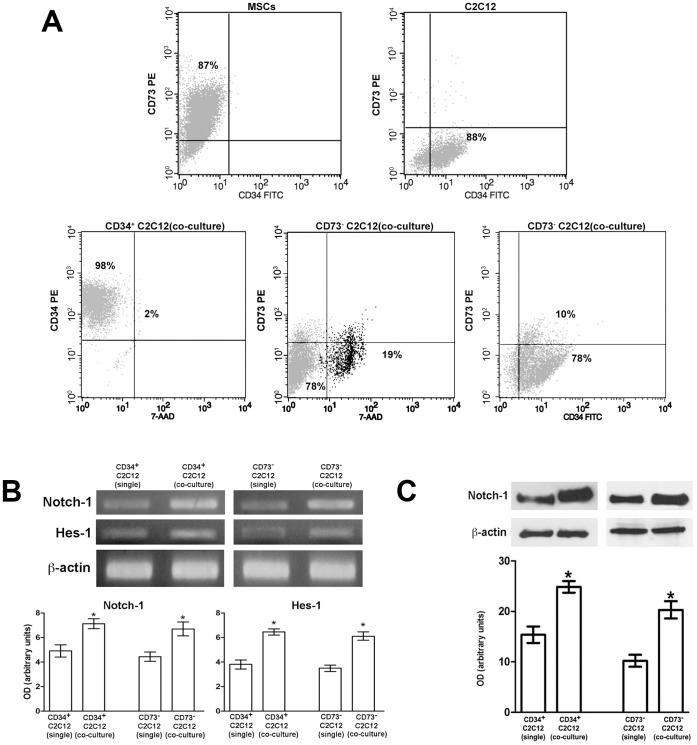
MSCs induce Notch-1 and Hes-1 expression in C2C12 myoblasts. A) Flow cytometric analysis of CD34 and CD73 antigen expression in MSCs and C2C12 cells in single cultures and in C2C12 myoblasts after isolation from MSCs in the co-culture by immunomagnetical separation using anti CD34 and anti CD73 antibodies (CD34^+^ C2C12; CD73^-^ C2C12). B,C) CD34^+^ C2C12 and CD73^-^ C2C12 cells in co-culture immunomagnetically isolated from MSCs were analyzed for the expression of Notch-1 and Hes-1 by RT-PCR (B) and Western Blotting (C). Note that Notch-1 and Hes-1 expression is robustly induced in the myoblastic cells after co-culturing with MSCs as compared with the single culture. Densitometric analysis of the bands normalized to β-actin is reported in the corresponding histograms. Data represent the results of at least three independent experiments with similar results. *p<0.05.

**Figure 5:**
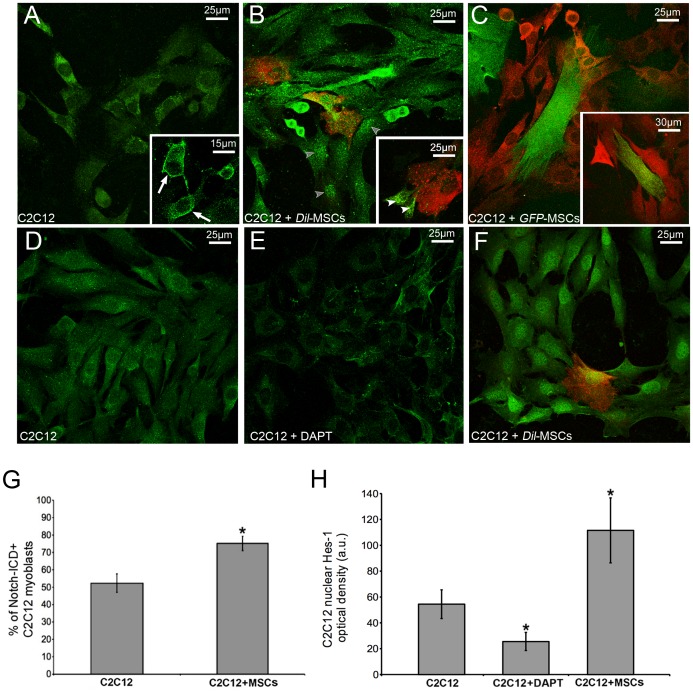
Notch-1 signaling is activated in C2C12 myoblasts upon co-culture with MSCs. Confocal immunofluorescence analysis of Notch-1 (A-C) and Hes-1 (D-F) expression in C2C12 cells in single and co-culture with *Dil*(red)- or *GFP*(green)-labeled MSCs for 24 h. After the co-culture, C2C12 cells reveal a stronger reactivity for the activated Notch-intracellular domain (Notch-ICD) and for Hes-1, which is visible inside the nucleus. As shown in the inserts, Notch-1 is preferentially located at the cell membrane (arrows) in the single cultured C2C12 cells, whereas it is found within the cytoplasm (white arrowheads) and nucleus (grey arrowheads) in the co-cultured cells. E) C2C12 myoblast were treated with 5 µM DAPT to inhibit Notch-1 activation and assayed for Hes-1 expression. The corresponding quantitative analysis is reported in the histograms (G,H). Data represent the results of at least three independent experiments with similar results. *p<0.05.

To isolate myoblasts from MSCs after co-culture, the cells were first assayed by flow cytometry to identify cell surface markers selectively expressed by one or the other cell types. We found that CD34 was expressed on C2C12 cells while was absent in MSCs, whereas the mesenchymal CD73 marker was present at high levels only in MSCs (Fig. 4A, upper panels; cell viability >90%). The cells in co-culture were then immunomagnetically separated using anti-CD34 and anti-CD73 PE-conjugated antibodies and anti-PE MicroBeads, and the recovered C2C12 cell fraction (Fig. 4A lower panels), CD34^+^(mean purity 98±2% and cell viability 90±7.2%) and CD73^-^ (mean purity 80.0±3% and cell viability 91.3±9.0%) were processed for total RNA and protein extraction. The results showed that 24 h of co-culture stimulated Notch-1 expression at the mRNA and protein levels in the myoblastic cells (Fig. 4B,C). The mRNA levels of Hes-1 were also up-regulated in the co-culture (Fig. 4B). By confocal microscopy, using a specific antiserum recognizing both Notch-1 receptor and its activated form, Notch-1 intracellular domain (Notch-ICD), we observed that co-cultured C2C12 cells expressed higher levels of cytoplasmic and nuclear Notch-ICD as compared with control cells (Fig. 5A–C,G). Moreover, it was possible to detect that the expression of Hes-1, increased in the co-cultured myoblasts and was predominantly localized within the nuclei (Fig. 5D–F,H).

### MSCs Influence C2C12 Cell Proliferation Through Paracrine Mechanisms

**Figure 6:**
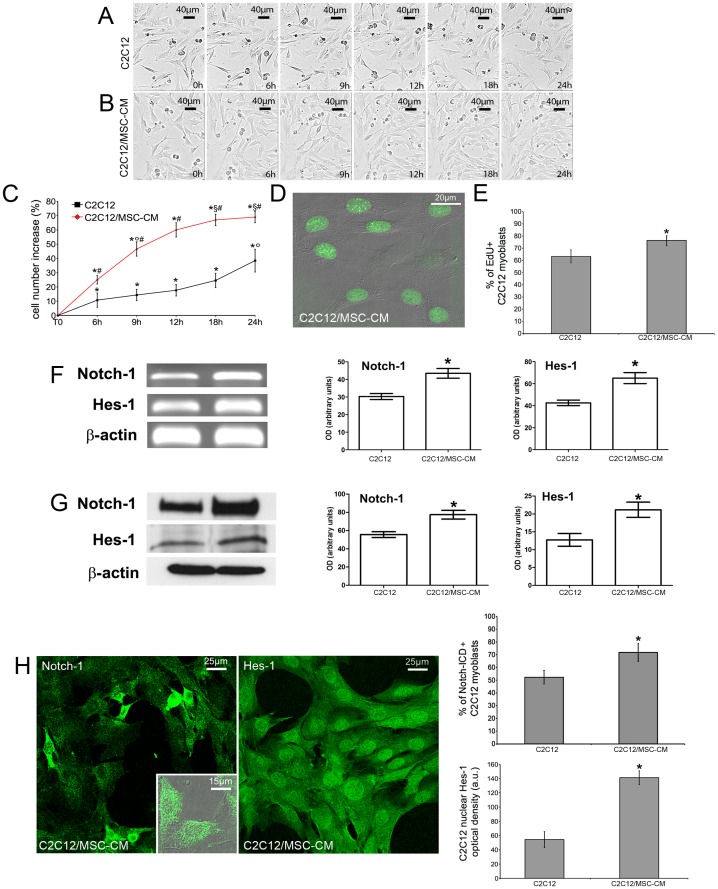
MSCs influence C2C12 myoblast proliferation through paracrine mechanisms. C2C12 cells were grown in single culture (C2C12) or exposed to MSC-derived CM (C2C12/MSC-CM) and their proliferative activity assessed by time lapse videomicroscopy (A-C), EdU (green) incorporation (D, E), Notch-1 and Hes-1 expression by RT-PCR (F), Western blotting (G) and confocal immunofluorescence (H). Quantitative analyses of the results shown are reported in the histograms. Data represent the results of at least three independent experiments with similar results. * *p<0.05* versus T0; ° *p<0.05* versus the earlier time points; § *p<0.05* vs 9 h; # *p<0.05* versus single culture. D,E: * *p<0.05*.

**Figure 7:**
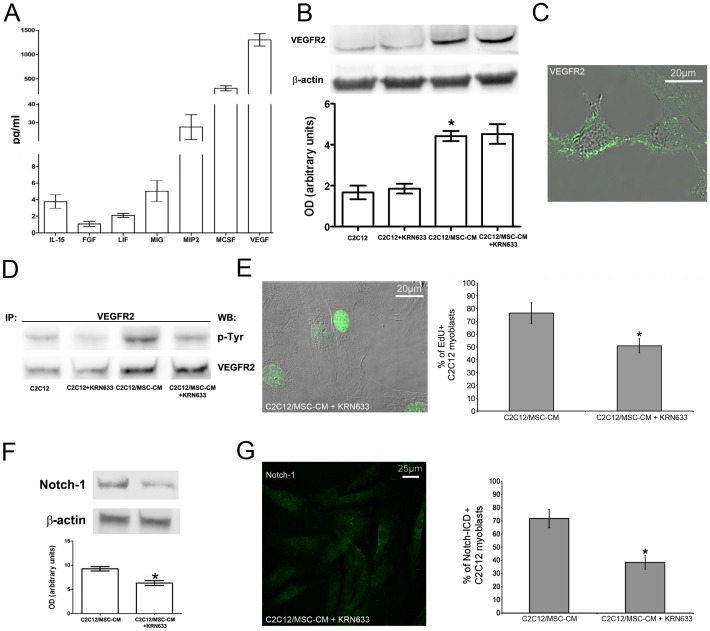
MSCs influence C2C12 myoblast proliferation through the release of VEGF. A) Cytokine and growth factor secretion profiles by MSCs grown in C2C12 differentiation medium (MSC-CM). B) Western Blotting analysis of VEGFR2 expression in C2C12 cells in single culture (C2C12) or exposed to MSC-CM (C2C12/MSC-CM), in the presence or absence of VEGFR2 inhibitor, KRN633. C) Superimposed DIC and fluorescence image showing cellular localization of VEGFR2 in C2C12 cells; the staining (green) is mainly localized at the cell surface. D) VEGFR2 phosphorylation in C2C12 cells in the noted experimental conditions, assayed by Western Blotting analysis performed on the immunoprecipitated VEGFR2 protein. Note that VEGFR2 expression and phosphorylation levels increase in the cells exposed to MSC-CM as compared with control. E) Superimposed DIC and fluorescence image showing nuclear EdU (green) staining and corresponding quantitative analysis. (F,G) Notch-1 expression by (F) Western blotting and (G) confocal immunofluorescence in C2C12 cells in the indicated experimental conditions. The quantitative analyses are reported in the histograms. Note that EdU staining and Notch-1 expression are significantly affected by treatment with the VEGFR2 inhibitor, KRN633. Data represent the results of at least three independent experiments with similar results. * *p<0.05.*

To discriminate the mechanisms underlying C2C12-MSC cell interaction, C2C12 myoblasts were cultured with MSC-CM for 24 h. Of interest, the stimulatory effect of MSC-CM on C2C12 cell growth approximated that of the co-cultures as judged by Time-lapse (Fig. 6A–C) and EdU incorporation (Fig. 6D,E) revealing that factors secreted by MSCs were mainly responsible for the observed enhancement of myoblast proliferation in the co-culture system. Consistent with this finding, the treatment with MSC-CM yielded similar results in term of Notch-1 and Hes-1 expression (Fig. 6F–H), thus suggesting that MSCs in co-culture behave as stromal supporting cells to positively influence the expansion of skeletal myoblasts in a paracrine manner. In order to better investigate this interaction, we searched for signals emanating from MSCs that could influence myoblast proliferation. We found that MSC-CM contained significant amount of cytokines and growth factors, particularly VEGF (Fig. 7A), whose signal potential has been previously shown to occur through Notch-1 pathway [28]. We, therefore, asked whether VEGF activity was required for MSCs to influence C2C12 cell growth. Our results showed that VEGFR2 (Flk-1), the major mediator of myoblast cell proliferation in response to VEGF [29], was expressed by C2C12 cells (Fig. 7B,C). By Western blot analysis with an anti-phospho-tyrosine antibody performed on the immunoprecipitated VEGFR2 (Flk-1) protein, we found that the levels of VEGFR2 tyrosine phosphorylation were significantly increased in the myoblastic cells cultured in MSC-CM as compared to controls (Fig. 7D). Interestingly, the treatment with the selective pharmacological VEGFR2 inhibitor, KRN633, markedly attenuated the receptor phosphorylation without modifying its expression (Fig. 7B, D) and reduced the effects of MSC–CM on Notch-1 activation and EdU incorporation (Fig. 7E–G) in C2C12 cells, demonstrating that VEGF could mediate the paracrine effects of MSCs on myoblast proliferation. Interestingly, the findings that VEGFR2 expression levels were increased in the C2C12 cells stimulated with MSC-CM (Fig. 7B) indicated that secreted factors by MSCs could also be able to modulate VEGF responsiveness in these cells.

**Figure 8:**
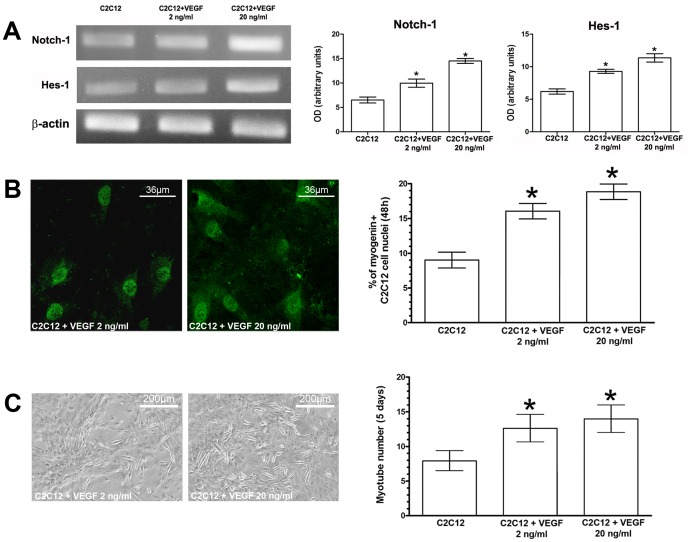
Effects of soluble VEGF on C2C12 myoblast proliferation and differentiation. C2C12 cells in single culture were treated with different concentrations of soluble VEGF and assayed for Notch-1 and Hes-1 expression by RT-PCR (A), myogenin expression (green) by confocal immunofluorescence (B), and for myotube formation by phase contrast microscopy (C). The quantitative analyses are reported in the histograms. Data represent the results of at least three independent experiments with similar results.* *p<0.05.*

Finally, the role of VEGF in C2C12 myoblast proliferation and differentiation was further assayed in experiments in which the single cultured cells were exposed to different concentrations (2 ng/ml and 20 ng/ml) of soluble VEGF (Fig. 8). As expected, the treatments stimulated both the expression of Notch-1 and Hes-1 within the first 24 h, and of myogenin in the later times (48 h) as well as increased the tendency of the myoblasts to fuse into multinucleated myotubes (5 days).

## Discussion

Growing evidence suggests that MSCs are attractive candidates for cell-based therapy in regenerative medicine, thanks to their trophic activity and immunosuppressive properties, which allow them to be used in different clinical contexts [19], [21]. In the present study, we offer evidence that these cells can establish complex paracrine interactions with skeletal myoblasts and stimulate their proliferation and differentiation through the up-regulation of Notch-1 signaling, a key regulator of muscle progenitor cell proliferation and myogenesis [27], [30]. In such a view, our data extend previous observations from our group on the remarkable ability of MSCs to engage in cross talk with stem/precursor cells [22] and provide novel clues for a role of these cells in the stimulation of the endogenous skeletal muscle repair/regeneration. In this line of thought, it is worth of noting that a close relationship between stromal and satellite cells has been shown within the skeletal muscle niche and a role for the stromal cells as a natural scaffold on which stem cells interact and proliferate, has been proposed [31], thus supporting the instructive potential of MSCs for the recruitment of the intrinsic muscle stem cell pool.

We have also shown that MSCs contributed to myoblast proliferation through the released of VEGF, on the basis that VEGFR2 showed increased tyrosine phosphorylation in C2C12 cells cultured in MSC-derived CM and that its specific pharmacological inhibition was able to significantly reduce the effects of this medium on C2C12 cell proliferation and Notch-1 activation. Of interest, VEGFR2 expression levels were dependent on the ligand availability, since they increased significantly in the cells stimulated with MSC-CM. Since previous observation have demonstrated that VEGF regulates VEGFR2 transcription by a positive feedback loop through its promoter region [32]–[34], it is possible that MSCs may be able not only to activate VEGF signaling, but also to modulate its responsiveness in the myoblastic cells.

Although VEGF has been originally described as a crucial regulator of vascular development during embryogenesis and blood vessel formation in adult life, there is evidence suggesting that its effects may extend to a variety of other cell types, including myoblast and skeletal muscle fibers [29], [35], [36]. However, despite the emerging concept that satellite and endothelial cells co-operatively interact during angio-myogenesis [37], [38] and that VEGF plays an essential role in this bidirectional interaction [39], the involvement of this growth factor on muscle repair/regeneration has been poorly characterized. Of interest, recent observations show that VEGF is up-regulated in hypertrophic and hypoxic myofibres [40], [41], providing the basis for considering VEGF as a multifaceted factor for muscle growth and survival. In such a view, our data contribute to acknowledge the role of this growth factor in skeletal muscle tissue and open the interesting possibility that MSCs, through the sustained secretion of VEGF, may serve to structure a regenerative microenvironment for damaged skeletal muscle, stimulating myoblast proliferation in concert with neo-angiogenesis. It could be expected that the combination of MSCs with tissue engineering technology, involving the use of tissue-specific scaffolds to prolong the survival and integration of the encased MSCs at the implantation sites, may greatly improve the therapeutic potentials of these cells and subsequent regeneration of skeletal muscle. Experiments are ongoing in our lab to test this hypothesis.

In conclusion, we have demonstrated that MSCs support myoblast proliferation and accelerate their differentiation, and underscored the cellular and molecular cross-talk between the two cell types. We also offer circumstantial evidence that the secretion of VEGF by MSCs may be one of the potential mechanism through which these cells influence the fate of skeletal myoblasts and provide experimental background for considering these cells as potential valid therapeutic tools for skeletal muscle disease.

The advantage of using MSC-based cell therapy over the exogenously administered VEGF may rely on the possibility to obtain local, constant and biologically effective levels of this growth factor in the contest of the regenerating muscle and to modulate and integrate its action with those of the other paracrine factors released *in situ* by the engrafted cells.
